# Single-Cell Transcriptome Analysis in Melanoma Using Network Embedding

**DOI:** 10.3389/fgene.2021.700036

**Published:** 2021-07-05

**Authors:** Liming Wang, Fangfang Liu, Longting Du, Guimin Qin

**Affiliations:** School of Computer Science and Technology, Xidian University, Xi’an, China

**Keywords:** single cell, melanoma, cell type, gene regulatory network, network embedding

## Abstract

Single-cell sequencing technology provides insights into the pathology of complex diseases like cancer. Here, we proposed a novel computational framework to explore the molecular mechanisms of cancer called melanoma. We first constructed a disease-specific cell–cell interaction network after data preprocessing and dimensionality reduction. Second, the features of cells in the cell–cell interaction network were learned by node2vec which is a graph embedding technology proposed previously. Then, consensus clusters were identified by considering different clustering algorithms. Finally, cell markers and cancer-related genes were further analyzed by integrating gene regulation pairs. We exploited our model on two independent datasets, which showed interesting results that the differences between clusters obtained by consensus clustering based on network embedding (CCNE) were observed obviously through visualization. For the KEGG pathway analysis of clusters, we found that all clusters are extremely related to MicroRNAs in cancer and HTLV-I infection, and the hub genes in cluster specific regulatory networks, i.e., *ETS1*, *TP53*, *E2F1*, and *GATA3* are highly associated with melanoma. Furthermore, our method can also be extended to other scRNA-seq data.

## Introduction

Melanoma is a malignant tumor that develops from melanocytes and is a complex multifactorial disease caused by the interaction between genetic susceptibility factors and environmental exposure (Rastrelli et al., 2014; Situm et al., 2014). In the past, many studies have been focused on the molecular mechanisms of melanoma, demonstrating that PI3K-Akt and MEK-ERK signaling pathways’ hyperactivation is highly correlated with the malignant transformation and progression of melanoma (Wei et al., 2019). Significant progress has been made in targeted therapies which aim to dampen these two signaling pathways, but melanoma is becoming increasingly resistant to these therapies (Johnson and Sosman, 2015; Luke et al., 2017). Understanding the pathogenesis of melanoma may overcome this obstacle.

There have been many significant studies based on bulk RNA-seq data of melanoma. Kunz et al. (2018) analyzed two transcriptomic types of melanocytic nevi and primary melanomas, and identified genetic characteristics and mechanisms of early and late stages of melanoma. By integrating transcriptomic and structural genomic data, Berger et al. (2010) identified 11 novel melanoma gene fusions and 12 novel readthrough transcripts resulting from basic genome rearrangements. But some limitations exist when only bulk RNA-seq data are available for the molecular expression level. The single-cell sequencing technology has brought new insights into complex biological phenomena. In particular, genome-wide single-cell measurements such as transcriptome sequencing can characterize cell composition and functional variation in homogeneous cell populations (Tang et al., 2009; Kolodziejczyk et al., 2015; Jaakkola et al., 2017). However, it is challenging to explore single-cell sequencing data effectively because of noise and dropout (Brennecke et al., 2013; Horvath et al., 2019; Bai et al., 2020). Researchers have begun to interpret the functional status of cancer cells at the single-cell level. Various computational methods have been used in cancer research and have helped the discovery of cancer development, metastasis, treatment resistance, and tumor microenvironment (Ren et al., 2018; Zhang et al., 2020). However, as far as we know, few studies focusing on molecular mechanisms of melanoma at the single-cell level (Fattore et al., 2019; Durante et al., 2020).

On the other hand, network embedding is becoming a powerful way of representing the features of nodes in complex networks, and is widely used in various fields, including medicine, biology, sociology, and finance. Several state-of-art network embedding algorithms are proposed to help accomplish analysis tasks of complex networks, e.g., DeepWalk (Perozzi et al., 2014) and node2vec (Grover and Leskovec, 2016). Network embedding is extremely useful for highly sparse single-cell sequencing data.

In this study, we applied network embedding to represent cell features, identified consensus clusters by consensus clustering, and investigated gene markers for melanoma. In the following section, we introduced the materials and methods in our model and then showed and analyzed the results. Finally, we made conclusions for our study.

## Materials and Methods

### Data Sources and Preprocessing

GSE72056 is an scRNA-seq data from Gene Expression Omnibus^1^ (Tirosh et al., 2016), which involves 19 melanoma patients, 23,686 genes, and 4,645 cells. After QC with Scater (McCarthy et al., 2017) and the batch effects elimination with Limma (Ritchie et al., 2015), we obtained 4,630 cells and 22,105 genes, respectively. Among the 4,630 cells, there are 1,346 malignant cells and 3,284 non-malignant cells. In our study, we only selected the malignant cells for further study. Then we performed feature selection with M3drop (Andrews and Hemberg, 2019) and obtained 6,786 genes eventually.

Another scRNA-seq data EXP0072 related to melanoma were downloaded from CancerSEA^2^ (Yuan et al., 2019), which was originally from GSE81383 (Gerber et al., 2017). EXP0072 contains the gene expression level of 307 cells and 18,938 genes. After QC, the batch effects elimination, and feature selection, there are 297 cells and 4,566 genes, respectively.

Gene regulation pairs were collected from the HTRIdb (Bovolenta et al., 2012) and TRRUST v2 (Han et al., 2018) databases. HTRIdb is an open-access TF-target gene interaction database that can be downloaded via a user-friendly web interface (Bovolenta et al., 2012). TRRUST is a TF-target interaction database for humans, and TRRUST v2 has a significant improvement from the first version of TRRUST, including a significantly increased size and a web interface (Han et al., 2018). There are 51,871 and 8,427 regulation pairs we collected from HTRIdb and TRRUST v2, respectively. The integrated regulation pairs are used as the source of regulation pairs.

### Consensus Clustering Based on Network Embedding (CCNE)

After preprocessing scRNA-seq data, we analyzed the data based on a network embedding model according to the following steps. It is worth noting that we log-transformed the data in QC of preprocess.

Step 1: Cell–cell interaction network construction

We used Euclidean distance to measure the interaction between cells because it treats the differences in different dimensions equally, and chose the average distance as the threshold to construct the cell–cell interaction network. Euclidean distance between two cells c1 and c2 is defined as follows:

(1) $$d\,i\,s\,t\,\left(X,Y\right)=\sqrt{\sum_{i=1}^{n}{\left(X_{i}-Y_{i}\right)}^{2}}$$

where *X* and *Y* are gene expression level vectors of c1 and c2, respectively, and *n* is the number of genes.

Step 2: Network embedding

Network embedding is a powerful measure in representing complex networks. Among these state-of-art network embedding algorithms, node2vec has been successfully used in many applications. Given any graph, it can learn the feature representation of nodes, which can then be used in various downstream machine learning tasks. In this algorithm, the parameters *p* and *q* can be flexibly changed to adjust the random walk strategy, which is helpful for the biased collection of node information (Grover and Leskovec, 2016).

Step 3: Consensus clustering

As there are various clustering algorithms, in this study, we tried to apply K-means (Macqueen, 1967), Gaussian mixture model (GMM) (Guorong et al., 2001), spectral clustering (Bach and Jordan, 2003), hierarchical clustering (Johnson, 1967), and Birch algorithm (Peng et al., 2018) to cluster the embedding vectors, and used the Silhouette Coefficients (Zhou and Gao, 2014) and Caliński–Harabaz score (Caliński and Harabasz et al., 1974) to evaluate the identified clusters. We determined the number of clusters *k* when the Silhouette Coefficient is optimal. The Silhouette Coefficients is a value from −1 to 1, and the larger the value, the better the result. So we selected the maximum Silhouette Coefficients value. Eventually, we chose three best algorithms for our data and constructed consensus clusters. Consensus clustering is defined as follows: we took the intersection of the clusters obtained from the three algorithms, and selected the result with the largest number of cells as the consensus clusters.

### Gene Regulatory Analysis

We selected the genes from each consensus clusters that expressed in more than 70% of cells; 70% of the gene expression is more representative, and the number of genes obtained by 70% is more appropriate, which is conducive to obtaining significant regulation pairs. Then we constructed the cluster specific regulatory networks according to the following procedure. First, we retained the regulation pairs in which the targets belong to the gene sets. Second, for each regulation pair, we calculated the similarity of the TF and its target. The similarity of gene regulation pair is defined as:

(2) $$s\,i\,m\,\left(g_{1},g_{2}\right)=1-\frac{\mathrm{dist}\,\left(g_{1},g_{2}\right)-\min\,\left(d\,i\,s\,t\right)}{\text{max}\,\left(d\,i\,s\,t\right)-\text{min}\,\left(d\,i\,s\,t\right)}$$

where *dist*(*g*_1_,*g*_2_) represents the Euclidean distance of the regulation pair (*g*_1_,*g*_2_), and *max*(*dist*) and *min*(*dist*) represent the maximum distance and the minimum distance in all regulation pairs, respectively. The larger the value, the stronger the correlation between the genes.

Then we identified the feed-forward loops (FFLs) (Jin, 2013) from these gene regulation pairs and constructed cluster specific gene regulatory networks by Cytoscape_v3.6.1 (Shannon et al., 2003). We took the top three genes with the highest degree from the specific gene regulatory network as hub genes.

## Results

**FIGURE 1:**
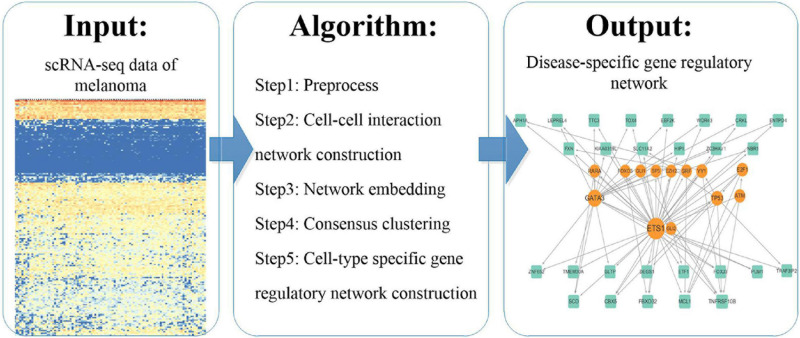
The pipeline of the study.

We analyzed scRNA-seq data according to the pipeline shown in Figure 1. We used existing methods to explore the molecular mechanisms of melanoma by this novel pipeline. We first preprocessed the scRNA-seq data and constructed a cell–cell interaction network based on Euclidean distance. And then we represented the cells using the network embedding algorithm node2vec and identified consensus clusters by consensus clustering. Finally, we constructed and analyzed cluster specific gene regulatory networks by integrating the expressed genes in scRNA-seq data and regulation pairs.

### Cell–Cell Interaction Network Construction and Network Embedding

We calculated the Euclidean distance between every pair of cells in GSE72056 and filtered the pairs with the distance less than the mean distance. As a result, we obtained a cell–cell interaction network with 1,312 nodes (cells) and 444,382 edges after discarding 34 isolated cells.

Then we applied node2vec to represent each cell as a vector in low-dimensional space. We selected the Silhouette Coefficients as the evaluation index and performed different hyperparameters from the set {0.25, 0.5, 1, 2, 4}. The hyperparameter with the maximum Silhouette Coefficients value is chosen. Finally, we chose *p* = 4 and *q* = 0.5 (Grover and Leskovec, 2016). As a result, we obtained the embedding vectors of the cells with 128 dimensions.

### Consensus Clustering and Clusters Identification

**FIGURE 2:**
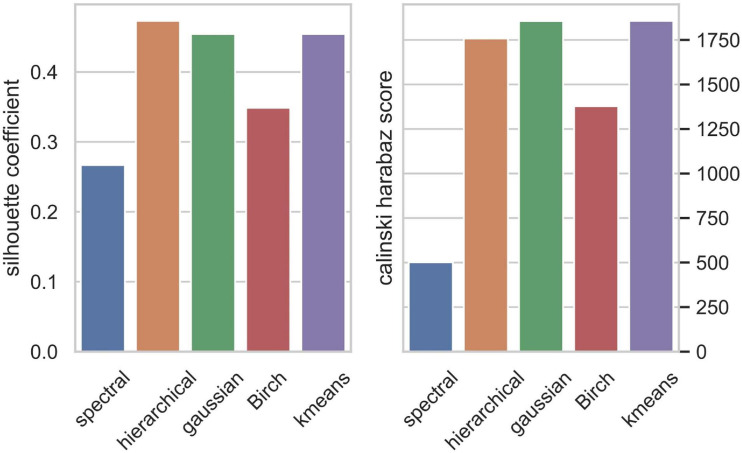
Clusters evaluation by Silhouette Coefficient **(left)** and Caliński–Harabaz score **(right)**.

**FIGURE 3:**
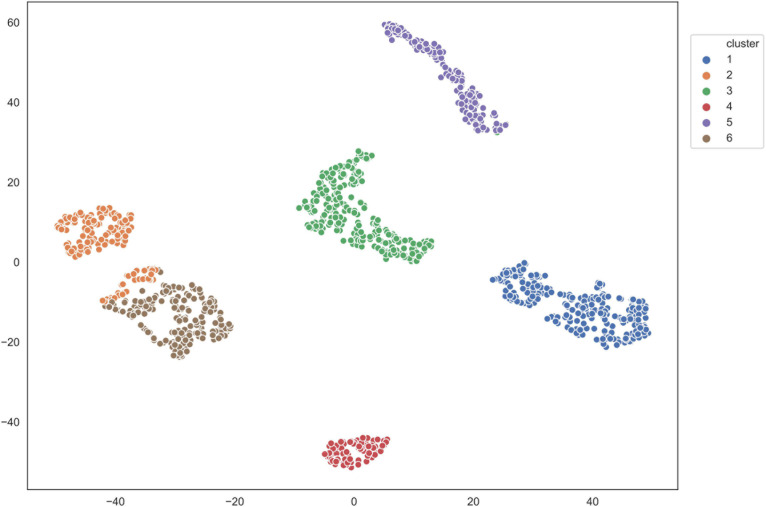
Visualization of clusters using T-SNE.

We applied the five clustering algorithms mentioned in Section “Materials and Methods” to cluster the embedding vectors (Table 1) and used the Silhouette Coefficient and Caliński–Harabaz score to evaluate the results (Figure 2). Among the five algorithms, Hierarchical clustering, GMM and K-means are significantly better than the other two methods according to the evaluation criteria, so we selected these three algorithms to obtain the consensus clusters. Finally, we obtained six clusters with 82, 120, 238, 222, 162, and 239 cells, respectively. It is worth noting that in the process of consensus clustering the cells belonging to different clusters resulted from different algorithms were discarded. We visualized the clusters using t-SNE (van der Maaten and Hinton, 2008), as shown in Figure 3.

**TABLE 1:** The number of cells in each cluster by different algorithms.

**The number of cells of cluster**	**1**	**2**	**3**	**4**	**5**	**6**
Spectral clustering	183	6	216	110	4	793
Hierarchical clustering	12	209	272	305	352	162
Gaussian mixture	111	240	238	287	178	258
Birch	554	11	223	127	220	177
K-means	246	258	238	298	94	178

### Gene Regulatory Network Construction and Analysis

**FIGURE 4:**
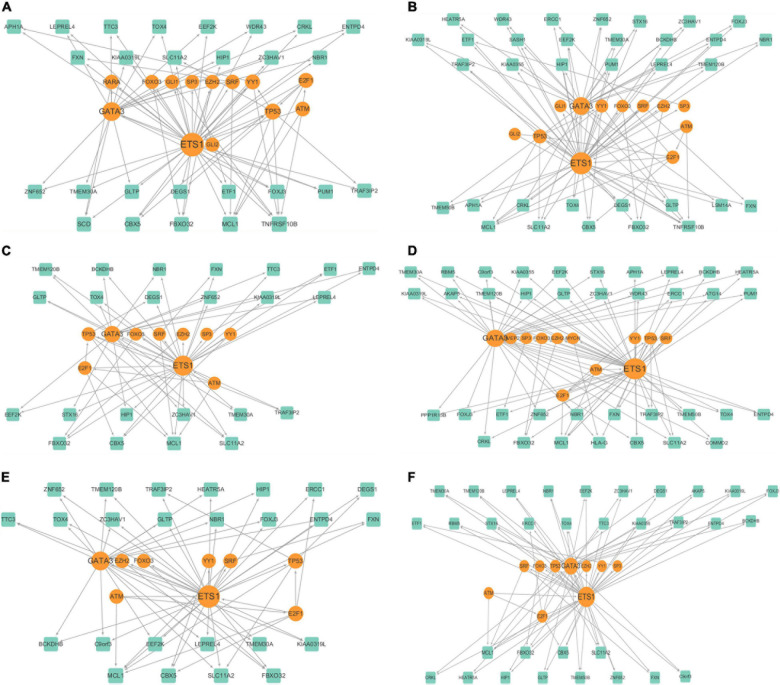
Cluster specific gene regulatory networks of **(A–F)** 6 clusters.

We filtered the genes that expressed in less than 70% of the cells in clusters, and identified 601, 729, 480, 744, 527, and 597 for the six clusters, respectively. Then for each cluster, we built a cluster specific regulatory network with the genes and discovered the FFLs. The information about the gene regulatory networks is shown in Table 2 and Figure 4.

**TABLE 2:** The number of regulation pairs in the cluster specific gene regulatory networks.

**Clusters**	**1**	**2**	**3**	**4**	**5**	**6**
TF-target	1,183	1,313	1,056	1,308	1,088	1170
Filtered TF-target	715	841	623	838	687	716
FFL	41	47	31	46	33	40

We further analyzed the cluster specific gene regulatory networks. We found that at least three genes existed in each cluster are known melanoma-related genes. Among them, *ATM*, *TP53*, and *FOXO3* belonged to each cluster. *ATM* serves as a regulator of a variety of downstream proteins, including tumor suppressor proteins (Sanders et al., 2020). *FOXO3* may well induce apoptosis in melanoma cells by the expression of requisite genes (Segura et al., 2009). We examined the degree distributions of both TF and genes in the six networks, which indicated that all of the gene regulatory networks were scale-free. Generally, TFs had significantly higher degrees than target genes in each network, indicated that there is a complex combination of TF coordinated regulation and gene multiplicity. There is a negative correlation between the topological coefficients and degrees of TF and genes, which shows that hub genes are the only shared neighbor of nodes with fewer links. *ETS1* and *GATA3* are hub genes in all of the six clusters, and *E2F1* in five of the six clusters, and *TP53* in one cluster.

These hub genes are closely related to cancer, and are candidate biomarkers for melanoma. *TP53* is a known tumor suppressor and relates to melanoma (Shain et al., 2015). *ETS1* encodes a member of the *ETS* transcription factor family, and these proteins act as transcriptional activators or repressors of many genes which are involved in stem cell development, cell senescence and death, and tumorigenesis (Gluck et al., 2019). *GATA3* belongs to the *GATA* family of transcription factors and is an important regulator of T cell development. It also plays an important role in endothelial cell biology (Terra et al., 2021). *E2F1* is a member of the *E2F* transcription factor family. The *E2F* family is very important for controlling the cell cycle and inhibiting tumor proteins, and it is also the target of small DNA tumor virus transforming proteins (Murphy et al., 2021; Rocca et al., 2019).

**FIGURE 5:**
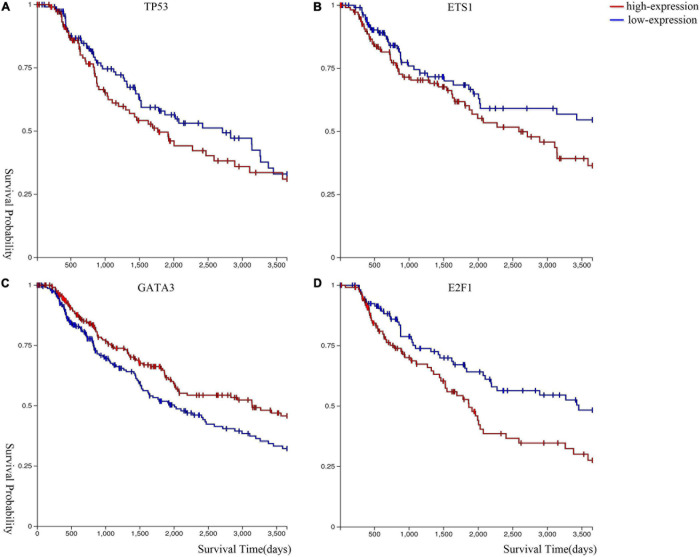
Kaplan–Meier survival curves of **(A)** *TP53*, **(B)** *ETS1*, **(C)** *GATA3*, and **(D)** *E2F1*.

We performed Kaplan–Meier survival analysis (Goldman et al., 2020) to analyze the expression level of these genes in melanoma. Figure 5 shows that melanoma patients with lower gene expression levels of *ETS1*, *TP53*, and *E2F1*, and higher gene expression level of *GATA3* have a higher survival probability.

We performed gene sets GO and pathway enrichment analysis using DAVID (DAVID Functional Annotation Bioinformatics Microarray Analysis^3^) (Huang et al., 2008; Huang da et al., 2009).

For GO enrichment analysis, we selected the top 10 items (PValue < 0.05) (Guo et al., 2020) for each cluster, and found that all clusters were associated with protein binding, nuclear chromatin, nucleoplasm and regulation of the apoptotic process. Cluster 1 and cluster 2 are more related to sequence-specific DNA binding and chromatin binding. Cluster 4 and cluster 6 are more associated with DNA binding, and cluster 5 and cluster 6 are relevant nuclear chromosome, telomeric region, and transcription factor binding. Also, cluster 2 is related to heart development, and cluster 3 is related to negative transcription regulation and DNA-templated.

**FIGURE 6:**
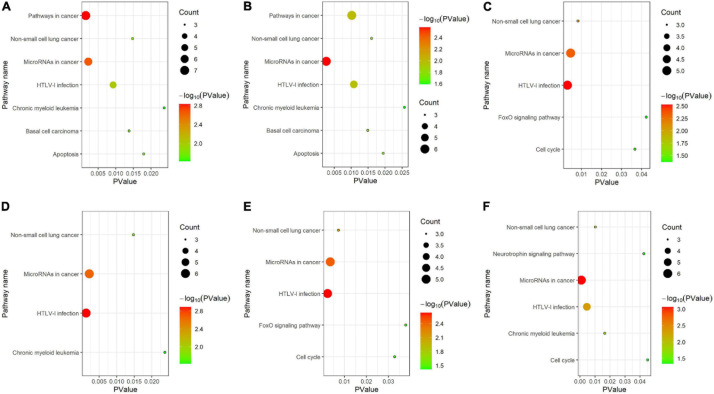
KEGG pathway analysis for **(A–F)** six clusters.

As shown in Figure 6, for KEGG pathway analysis, we chose all results with PValue < 0.05. From that, we got that all clusters are associated with MicroRNAs in cancer and HTLV-I infection. Cluster 1 and cluster 2 are more related to pathways in cancer, basal cell carcinoma and apoptosis, cluster 3 and cluster 5 are more related to FoxO signaling pathway, and cluster 6 is strongly associated with neurotrophic signaling pathway.

### Experiments on an Independent Dataset

We analyzed an independent dataset EXP0072 to verify our approach. We expressed the embedding vectors of 307 cells with 128 dimensions, which is the same as our previous experiment. The identified three clusters are shown in Supplementary Table 1 and Supplementary Figure 1. By consensus clustering, the numbers of cells were 71, 174, and 52 of the consensus clusters.

The numbers of genes that expressed in less than 70% of the cells in clusters were 220, 255, and 176, respectively. Then we built cluster specific gene regulatory networks accordingly. The information of cluster specific gene regulatory networks is shown in Supplementary Table 2 and Supplementary Figure 2.

Finally, we selected the top three genes with higher degrees (hub genes) for Kaplan–Meier survival analysis (Goldman et al., 2020) to test their functions in melanoma, which are shown in Supplementary Figure 3. These genes have a greater impact on the survival rate of melanoma. *E2F1* was the hub gene in both datasets. ESR1 encodes an estrogen receptor and ligand-activated transcription factor. The protein encoded by this gene regulates the transcription of many estrogen-inducible genes and is expressed in many non-reproductive tissues. *NF-kappa-B* is a transcription factor formed by the combination of *NFKB1* and *RELA*, which participates in many biological processes. *E2F4* encoded by this gene is a member of the E2F family of transcription factors. The E2F family plays a crucial role in the control of cell cycle and action of tumor suppressor proteins and is also a target of the transforming proteins of small DNA tumor viruses. *MYC* is a proto-oncogene and encodes a nuclear phosphoprotein that plays a role in cell cycle progression, apoptosis, and cellular transformation. Amplification of this gene is frequently observed in numerous human cancers (Murphy et al., 2021).

From the experiments, we found that the size of the dataset has impact on the computational cost, but not affects the network embedding, clustering, or quality of the reconstructed GRNs.

## Conclusion

We investigated the pathology of melanoma via scRNA-seq, which revealed the significant impact of hub genes in the development of melanoma. By constructing the cell–cell interaction network and obtaining the consensus clusters, we found that the differences between clusters are obvious. Through our further analysis of each cluster, we found the hub genes *ETS1*, *TP53*, *E2F1*, and *GATA3* are related to melanoma. At the same time, in order to verify the scalability of the method, we analyzed an independent melanoma dataset that the clusters were highly consistent, and hub genes have a great impact on melanoma.

## Data Availability Statement

The data underlying this article are available in Gene Expression Omnibus (http://www.ncbi.nlm.nih.gov/geo/) and CancerSEA at http://biocc.hrbmu.edu.cn/CancerSEA/goDownload.

## Author Contributions

GQ, FL, and LD conceived and developed the framework for consensus clusters identification and wrote this manuscript. LW and GQ provided important feedback in the framework process and edited the manuscript. All authors made significant contributions to the completion and writing of this manuscript, and read and approved the final manuscript.

## Conflict of Interest

The authors declare that the research was conducted in the absence of any commercial or financial relationships that could be construed as a potential conflict of interest.

## Footnotes

1 http://www.ncbi.nlm.nih.gov/geo/

2 http://biocc.hrbmu.edu.cn/CancerSEA/goDownload

3 ncifcrf.gov

## References

[B1] Andrews T. S. Hemberg M. (2019). M3Drop: dropout-based feature selection for scRNASeq. *Bioinformatics* 35 2865–2867. 30590489 10.1093/bioinformatics/bty1044 PMC6691329

[B2] Bach F. R. Jordan M. I. (2003). *Learning Spectral Clustering*, Vol. 16. Berkeley, CA: University of California.

[B3] Bai Y.-L. Baddoo M. Flemington E. K. Nakhoul H. N. Liu Y.-Z. (2020). Screen technical noise in single cell RNA sequencing data. *Genomics* 112 346–355. 30802598 10.1016/j.ygeno.2019.02.014

[B4] Berger M. F. Levin J. Z. Vijayendran K. Sivachenko A. Adiconis X. Maguire J. (2010). Integrative analysis of the melanoma transcriptome. *Genome Res.* 20 413–427. 20179022 10.1101/gr.103697.109 PMC2847744

[B5] Bovolenta L. A. Acencio M. L. Lemke N. (2012). HTRIdb: an open-access database for experimentally verified human transcriptional regulation interactions. *BMC Genomics* 13:405. 10.1186/1471-2164-13-405 22900683 PMC3472291

[B6] Brennecke P. Anders S. Kim J. K. Kołodziejczyk A. A. Zhang X. Proserpio V. (2013). Accounting for technical noise in single-cell RNA-seq experiments. *Nat. Methods* 10 1093–1095. 24056876 10.1038/nmeth.2645

[B7] Caliński T. Harabasz J. (1974). A dendrite method for cluster analysis. *Commun. Stat.* 3 1–27.

[B8] Durante M. A. Rodriguez D. A. Kurtenbach S. Kuznetsov J. N. Sanchez M. I. Decatur C. L. (2020). Single-cell analysis reveals new evolutionary complexity in uveal melanoma. *Nat. Commun.* 11:496. 31980621 10.1038/s41467-019-14256-1 PMC6981133

[B9] Fattore L. Ruggiero C. F. Liguoro D. Mancini R. Ciliberto G. (2019). Single cell analysis to dissect molecular heterogeneity and disease evolution in metastatic melanoma. *Cell Death Dis.* 10:827. 31672982 10.1038/s41419-019-2048-5 PMC6823362

[B10] Gerber T. Willscher E. Loeffler-Wirth H. Hopp L. Schadendorf D. Schartl M. (2017). Mapping heterogeneity in patient-derived melanoma cultures by single-cell RNA-seq. *Oncotarget* 8 846–862. 27903987 10.18632/oncotarget.13666 PMC5352202

[B11] Gluck C. Glathar A. Tsompana M. Nowak N. Garrett-Sinha L. A. Buck M. J. (2019). Molecular dissection of the oncogenic role of ETS1 in the mesenchymal subtypes of head and neck squamous cell carcinoma. *PLoS Genet.* 15:e1008250. 10.1371/journal.pgen.1008250 31306413 PMC6657958

[B12] Goldman M. J. Craft B. Hastie M. Repecka K. McDade F. Kamath A. (2020). Visualizing and interpreting cancer genomics data via the Xena platform. *Nat. Biotechnol.* 38 675–678. 32444850 10.1038/s41587-020-0546-8 PMC7386072

[B13] Grover A. Leskovec J. (2016). “node2vec: scalable feature learning for networks,” in *KDD ′ 16: Proceedings of the 22nd ACM SIGKDD International Conference on Knowledge Discovery and Data Mining*, Vol. 2016 (Newyork, NY: ACM), 855–864. 10.1145/2939672.2939754 PMC5108654 27853626

[B14] Guo Q. Y. Wang J. W. Gao Y. Li X. Hao Y. Y. Ning S. W. (2020). Dynamic TF-lncRNA regulatory networks revealed prognostic signatures in the development of ovarian cancer. *Front. Bioeng. Biotech.* 8:460. 10.3389/fbioe.2020.00460 32478062 PMC7237576

[B15] Guorong X. Wei Z. Peiqi C. (2001). “EM algorithms of Gaussian mixture model and hidden Markov model,” in *Proceedings 2001 International Conference on Image Processing (Cat No01CH37205)*, (Thessaloniki: IEEE).

[B16] Han H. Cho J.-W. Lee S. Yun A. Kim H. Bae D. (2018). TRRUST v2: an expanded reference database of human and mouse transcriptional regulatory interactions. *Nucleic Acids Res.* 46 D380–D386. 29087512 10.1093/nar/gkx1013 PMC5753191

[B17] Horvath R. Laenen B. Takuno S. Slotte T. (2019). Single-cell expression noise and gene-body methylation in Arabidopsis thaliana. *Heredity* 123 81–91. 30651589 10.1038/s41437-018-0181-z PMC6781109

[B18] Huang D. W. Sherman B. T. Lempicki R. A. (2008). Systematic and integrative analysis of large gene lists using DAVID bioinformatics resources. *Nat. Protoc.* 4 44–57. 10.1038/nprot.2008.211 19131956

[B19] Huang da W. Sherman B. T. Lempicki R. A. (2009). Bioinformatics enrichment tools: paths toward the comprehensive functional analysis of large gene lists. *Nucleic Acids Res.* 37 1–13. 19033363 10.1093/nar/gkn923 PMC2615629

[B20] Jaakkola M. K. Seyednasrollah F. Mehmood A. Elo L. L. (2017). Comparison of methods to detect differentially expressed genes between single-cell populations. *Brief. Bioinform.* 18 735–743. 27373736 10.1093/bib/bbw057 PMC5862313

[B21] Jin G. (2013). “Feed forward loop,” in *Encyclopedia of Systems Biology*, eds Dubitzky W. Wolkenhauer O. Cho K.-H. Yokota H. (New York, NY: Springer), 737–738.

[B22] Johnson D. B. Sosman J. A. (2015). Therapeutic advances and treatment options in metastatic melanoma. *JAMA Oncol.* 1 380–386. 26181188 10.1001/jamaoncol.2015.0565

[B23] Johnson S. C. (1967). Hierarchical clustering schemes. *Psychometrika* 32 241–254. 5234703 10.1007/BF02289588

[B24] Kolodziejczyk A. A. Kim J. K. Svensson V. Marioni J. C. Teichmann S. A. (2015). The technology and biology of single-cell RNA sequencing. *Mol. Cell* 58 610–620. 26000846 10.1016/j.molcel.2015.04.005

[B25] Kunz M. Loffler-Wirth H. Dannemann M. Willscher E. Doose G. Kelso J. (2018). RNA-seq analysis identifies different transcriptomic types and developmental trajectories of primary melanomas. *Oncogene* 37 6136–6151. 29995873 10.1038/s41388-018-0385-y

[B26] Luke J. J. Flaherty K. T. Ribas A. Long G. V. (2017). Targeted agents and immunotherapies: optimizing outcomes in melanoma. *Nat. Rev. Clin. Oncol.* 14 463–482. 28374786 10.1038/nrclinonc.2017.43

[B27] Macqueen J. (1967). “Some methods for classification and analysis of multivariate observations,” in *Proceedings of the 5th Berkeley Symposium on Mathematical Statistics and Probability*, Vol. 1 (Berkeley, CA: University of California Press), 281–297.

[B28] McCarthy D. J. Campbell K. R. Lun A. T. L. Wills Q. F. (2017). Scater: pre-processing, quality control, normalization and visualization of single-cell RNA-seq data in R. *Bioinformatics* 33 1179–1186. 28088763 10.1093/bioinformatics/btw777 PMC5408845

[B29] Murphy M. Brown G. Wallin C. Tatusova T. Pruitt K. Murphy T. (2021). “Gene help: integrated access to genes of genomes in the reference sequence collection,” in *Gene Help [Internet]*, (Bethesda, MD: National Center for Biotechnology Information (US)). Available online at: https://www.ncbi.nlm.nih.gov/books/NBK3841/ (accessed February 1, 2021).

[B30] Peng K. Zheng L. Xu X. Lin T. Leung V. C. M. (2018). *Balanced Iterative Reducing and Clustering Using Hierarchies with Principal Component Analysis (PBirch) for Intrusion Detection over Big Data in Mobile Cloud Environment.* Cham: Springer International Publishing.

[B31] Perozzi B. Al-Rfou R. Skiena S. (2014). “DeepWalk: online learning of social representations,” in *Proceedings of the 20th ACM SIGKDD International Conference On Knowledge Discovery And Data Mining*, (New York, NY: Association for Computing Machinery), 701–710.

[B32] Rastrelli M. Tropea S. Rossi C. R. Alaibac M. (2014). Melanoma: epidemiology, risk factors, pathogenesis, diagnosis and classification. *In Vivo* 28 1005–1011. 25398793

[B33] Ren X. Kang B. Zhang Z. (2018). Understanding tumor ecosystems by single-cell sequencing: promises and limitations. *Genome Biol.* 19:211. 30509292 10.1186/s13059-018-1593-z PMC6276232

[B34] Ritchie M. E. Phipson B. Wu D. Hu Y. Law C. W. Shi W. (2015). limma powers differential expression analyses for RNA-sequencing and microarray studies. *Nucleic Acids Res.* 43:e47. 25605792 10.1093/nar/gkv007 PMC4402510

[B35] Rocca M. S. Benna C. Mocellin S. Rossi C. R. Msaki A. Di Nisio A. (2019). E2F1 germline copy number variations and melanoma susceptibility. *J. Transl. Med.* 17:181. 31142321 10.1186/s12967-019-1933-0 PMC6542053

[B36] Sanders J. T. Freeman T. F. Xu Y. Golloshi R. Stallard M. A. Hill A. M. (2020). Radiation-induced DNA damage and repair effects on 3D genome organization. *Nat. Commun.* 11:6178. 33268790 10.1038/s41467-020-20047-w PMC7710719

[B37] Segura M. F. Hanniford D. Menendez S. Reavie L. Zou X. Alvarez-Diaz S. (2009). Aberrant miR-182 expression promotes melanoma metastasis by repressing FOXO3 and microphthalmia-associated transcription factor. *Proc. Natl. Acad. Sci. U. S. A.* 106 1814–1819. 19188590 10.1073/pnas.0808263106 PMC2634798

[B38] Shain A. H. Yeh I. Kovalyshyn I. Sriharan A. Talevich E. Gagnon A. (2015). The genetic evolution of melanoma from precursor lesions. *N. Engl. J. Med.* 373 1926–1936. 26559571 10.1056/NEJMoa1502583

[B39] Shannon P. Markiel A. Ozier O. Baliga N. S. Wang J. T. Ramage D. (2003). Cytoscape: a software environment for integrated models of biomolecular interaction networks. *Genome Res.* 13 2498–2504. 14597658 10.1101/gr.1239303 PMC403769

[B40] Situm M. Buljan M. Kolic M. Vucic M. (2014). Melanoma – clinical, dermatoscopical, and histopathological morphological characteristics. *Acta Dermatovenerol. Croat.* 22 1–12. 24813835

[B41] Tang F. Barbacioru C. Wang Y. Nordman E. Lee C. Xu N. (2009). mRNA-Seq whole-transcriptome analysis of a single cell. *Nat. Methods* 6 377–382. 19349980 10.1038/nmeth.1315

[B42] Terra S. Roden A. C. Aubry M. C. Yi E. S. J. Boland J. M. (2021). Utility of immunohistochemistry for MUC4 and GATA3 to aid in the distinction of pleural sarcomatoid mesothelioma from pulmonary sarcomatoid carcinoma. *Arch. Pathol. Lab. Med.* 145 208–213. 33501493 10.5858/arpa.2019-0647-OA

[B43] Tirosh I. Izar B. Prakadan S. M. Wadsworth M. H. II Treacy D. Trombetta J. J. (2016). Dissecting the multicellular ecosystem of metastatic melanoma by single-cell RNA-seq. *Science* 352 189–196. 27124452 10.1126/science.aad0501 PMC4944528

[B44] van der Maaten L. Hinton G. (2008). Visualizing data using t-SNE. *J. Mach. Learn. Res.* 9 2579–2605.

[B45] Wei X. Gu X. Ma M. Lou C. (2019). Long noncoding RNA HCP5 suppresses skin cutaneous melanoma development by regulating RARRES3 gene expression via sponging miR-12. *Onco Targets Ther.* 12 6323–6335. 31496735 10.2147/OTT.S195796 PMC6698080

[B46] Yuan H. T. Yan M. Zhang G. X. Liu W. Deng C. Y. Liao G. M. (2019). CancerSEA: a cancer single-cell state atlas. *Nucleic Acids Res.* 47 D900–D908. 30329142 10.1093/nar/gky939 PMC6324047

[B47] Zhang J. Guan M. Wang Q. Zhang J. Zhou T. Sun X. (2020). Single-cell transcriptome-based multilayer network biomarker for predicting prognosis and therapeutic response of gliomas. *Brief. Bioinform.* 21 1080–1097. 31329830 10.1093/bib/bbz040

[B48] Zhou H. B. Gao J. T. (2014). Automatic method for determining cluster number based on silhouette coefficient. *Adv. Mater. Res.* 951 227–230.

