# Supplementary material for: Single-Cell Transcriptome Analysis in Melanoma Using Network Embedding
Source: Front Genet. 2021 Jul 5;12:700036. doi: 10.3389/fgene.2021.700036 (PMC8287331; doi:10.3389/fgene.2021.700036)
Supplement: Supplementary file 1 [file Data_Sheet_1.docx]

**Figure S1.** Clusters were evaluated via Silhouette Coefficients and Calinski-Harabaz scores.


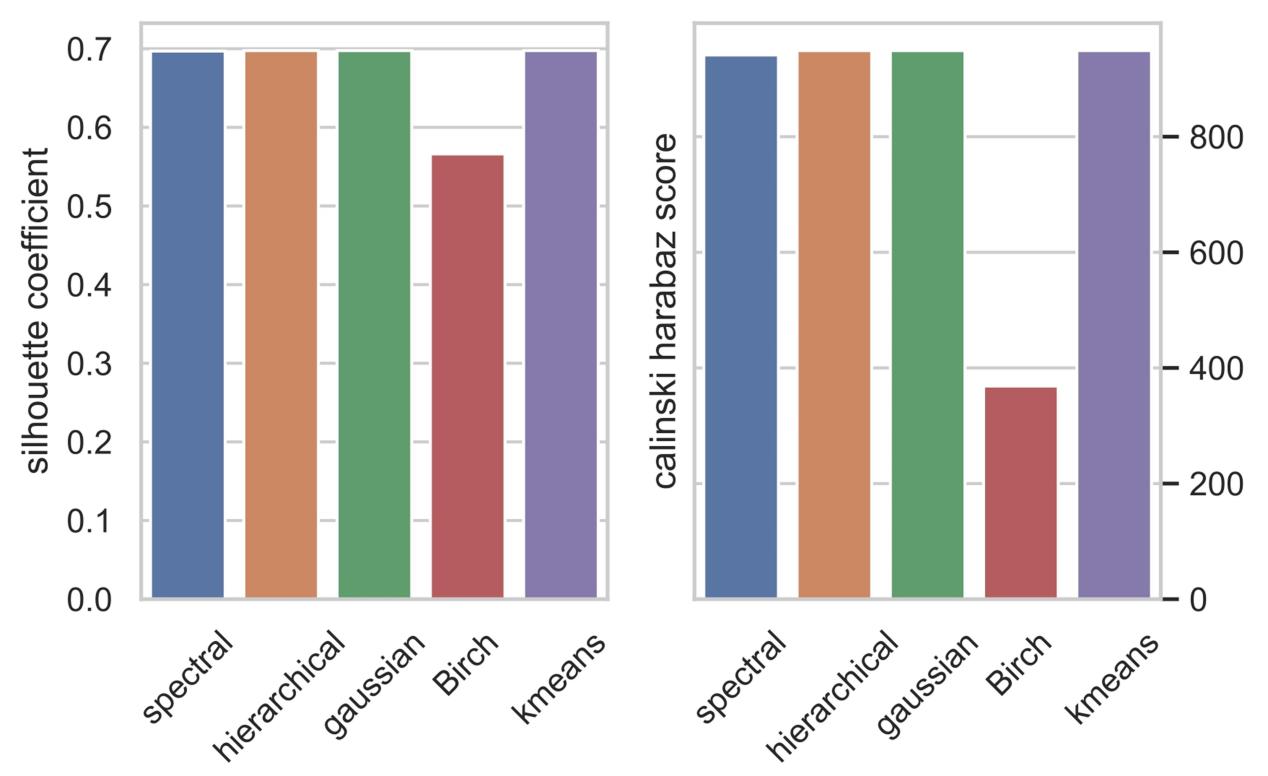


**Figure S2.** Cluster specific gene regulatory networks.


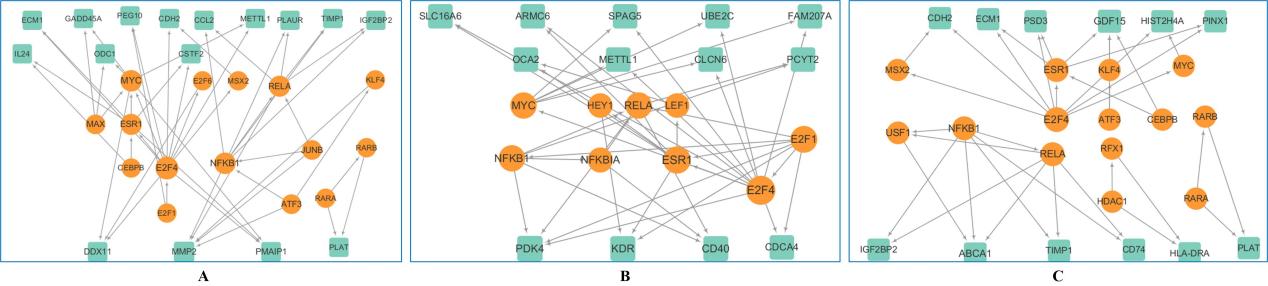


**Figure S3.** Kaplan-Meier survival curves of *E2F1*, *E2F4*, *ERS1*, *MYC*, *NFKB1*, *RELA*.


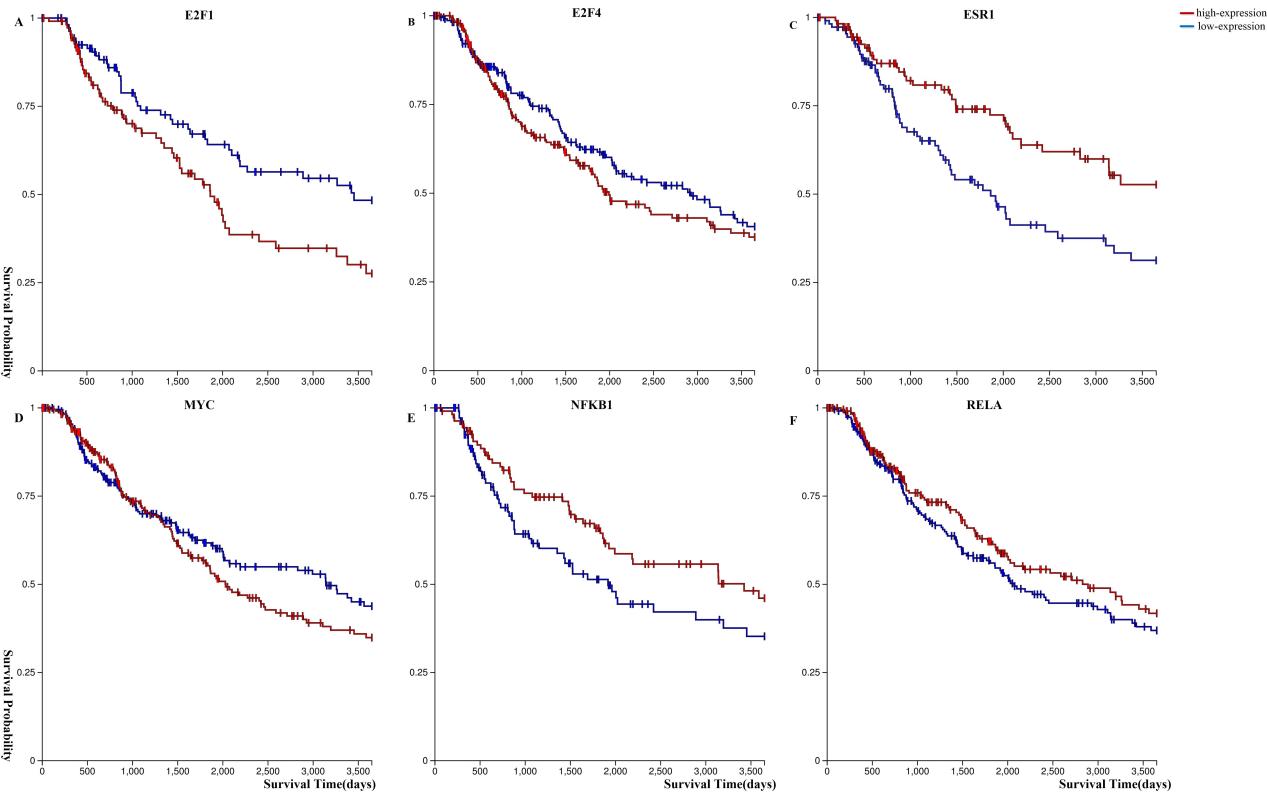


**Table S1.** Information of clusters identified in EXP0072 by different algorithms.

| The number of cells of clusters | 1 | 2 | 3 |
| --- | --- | --- | --- |
| Spectral Clustering | 175 | 71 | 51 |
| Hierarchical Clustering | 52 | 71 | 174 |
| Gaussian Mixture | 71 | 174 | 52 |
| Birch | 212 | 71 | 14 |
| K-means | 174 | 71 | 52 |

**Table S2.** The number of regulation pairs in different clusters.

| clusters | 1 | 2 | 3 |
| --- | --- | --- | --- |
| TF-target | 480 | 429 | 418 |
| Filtered TF-target | 449 | 394 | 405 |
| FFL | 23 | 19 | 15 |
